# Reviews

**Published:** 1886-01

**Authors:** 


### REVIEWS.

La Pathologie Comparée. Par le Dr. O. Larche. Paris, 1885.

The above is the title of an article taken from the "Dictionnaire Encyclopedique des Sciences Medicales," and published in pamphlet form.

The author calls attention to the advantages which the natural sciences have derived from the comparative method and to its well recognized utility in all branches of natural history, and proceeds to demonstrate its immense value in aiding pathological research.

The idea is not new, he says, but every day, in proportion as science progresses, observers are more and more convinced of its importance.

He appeals to the veterinarian to pursue his labors in observing and experimenting in the lower animals, thereby aiding by their results the efforts of their brethren in human medicine in obtaining a more complete knowledge of disease, thus repaying, in a measure, what they originally obtained from them.

The advantages of zoölogical gardens are dwelt on, and he urges the directors of them to make post-mortem examinations and dissect whenever an opportunity occurs. The value of experimental pathology is pointed out as shown by the labors of Pasteur, Burdon, Sanderson, Brown-Sequard, and others.

A brief history is given of comparative science, the best authors mentioned, its societies and museums, and the periodicals devoted to it. Of one of the latter he says: "Le Journal of Comparative Medicine and Surgery, qui paraît à New York, depuis 1880, est un Journal de M dicine Véterinaire, dans lequel une large place est dounée à la relation de faits observés sur d'autres animaux que ceux dont s'occupent specialement les véterinaires."

Reference is also made to articles on the subject by Dr. T. E. Satterthwaite.

The pamphlet contains thirty pages of interesting matter, which will well repay a veterinarian to peruse. J. O'L.

Post-mortem Examinations. By Prof. Rudolph Virchow. Translated by T. P. Smith, M.D. Philadelphia: P. Blakiston, Son & Co., 1885.

Here is to be found condensed in 140 pages, the early experience in autopsies of the most eminent modern pathologist, and his successful endeavor to develop a systematic method of conducting them. To the medical jurist it is invaluable, and its perusal will enable him to avoid embarrassing mistakes in noting post-mortem appearances. The veterinarian will find in this little book many valuable suggestions which will be of service to him in his dissections.

Physicians' Visiting List, 1886. Lindsay & Blakiston, Philadelphia.

This little book, now in its thirty-fifth year, is certainly the best Visiting List published. The present volume keeps up the high reputation sustained in former years, and needs no further commendation from us.

The Physicians' Magazine, published in Philadelphia, has taken up the subject of State Medicine, giving a synopsis of the annual report to the American Academy of Medicine, by Drs. R. J, Dunglison and H. O. Marcy, committee (read at the annual meeting at New York, October 29, 1885).

In this report a comprehensive statement of the actual progress made in the direction of medical legislation will be found. It contains the opinions on the subject of members of the medical profession in all parts of the United States—men who have taken an active part in protecting the public from quackery and fraud, and whose high professsional standing cannot fail to secure them a hearing in such important matters as those relating to human life and health. This subject has been ably treated by Dr. F. S. Billings, in a paper read before the meeting of the medical editors of the United States at New Orleans, and published in this JOURNAL for July, 1885

TRANSACTIONS OF THE STATE MEDICAL ASSOCIATION OF TEXAS, 1885.—The Seventeenth Annual Session of the above association was held in Houston in the month of April, 1885. The time has gone by when the expression "Gone to Texas," implied a benighted condition of that region. Now the *Lone Star* has taken her place with her sister States in the path of progress. As might be expected our professional brethren are not behindhand, and the book before us, judging from the address of the President and papers read, proves that they are doing good work in the interests of medical science.

